# Land claim and loss of tidal flats in the Yangtze Estuary

**DOI:** 10.1038/srep24018

**Published:** 2016-04-01

**Authors:** Ying Chen, Jinwei Dong, Xiangming Xiao, Min Zhang, Bo Tian, Yunxuan Zhou, Bo Li, Zhijun Ma

**Affiliations:** 1 Coastal Ecosystems Research Station of Yangtze Estuary, Ministry of Education Key Laboratory for Biodiversity Science and Ecological Engineering, Institute of Biodiversity Science, Fudan University, Shanghai, 200438, China; 2 Department of Microbiology and Plant Biology, Center for Spatial Analysis, University of Oklahoma, Norman, OK 73019, USA; 3 State Key Laboratory of Estuary and Coastal Research, East China Normal University, Shanghai, 200062, China

## Abstract

Tidal flats play a critical role in supporting biodiversity and in providing ecosystem services but are rapidly disappearing because of human activities. The Yangtze Estuary is one of the world’s largest alluvial estuaries and is adjacent to the most developed economic zone in China. Using the Yangtze Estuary as a study region, we developed an automatic algorithm to estimate tidal flat areas based on the Land Surface Water Index and the Normalized Difference Vegetation Index. The total area of tidal flats in the Yangtze Estuary has decreased by 36% over the past three decades, including a 38% reduction in saltmarshes and a 31% reduction in barren mudflats. Meanwhile, land claim has accumulated to 1077 km^2^, a value that exceeds the area of the remaining tidal flats. We divided the Yangtze Estuary into Shanghai and Jiangsu areas, which differ in riverine sediment supply and tidal flat management patterns. Although land claim has accelerated in both areas, the decline in tidal flat area has been much greater in Jiangsu than in Shanghai because of abundant supplies of sediment and artificial siltation in the latter area. The results highlight the need for better coastal planning and management based on tidal flat dynamics.

Tidal flats, which are those areas between land and sea, include a variety of habitat types that in temperate zones can be divided into vegetated saltmarsh and non-vegetated mudflat. Nourished by the exchange of materials between land masses and oceans, tidal flats provide many crucial ecosystem services. For instance, they provide feeding grounds for migrating birds^1^, spawning and nursery habitats for fishes, and defense against storm surges. They also filter pollutants, promote nutrient cycling and carbon storage, and are used for recreation, tourism, and education. Tidal flats can also be claimed for commercial and residential use^2^. Tidal flats, however, are among the world’s most vulnerable ecosystems, having lost more than 50% and as much as 80% of their original area in many regions worldwide^3^. Tidal flats have been lost because of global factors like sea-level rise^4^, and also because of local factors such as coastal land claim^5^, reduction of riverine sediment^6^, and land compaction and subsidence^7^. The conservation and sustainable use of tidal flats would be facilitated by the development of an efficient tool for determining the temporal and spatial changes in tidal flat area. Such a tool would be useful for a broad range of investigations undertaken by coastal scientists, engineers, and managers^8^.

Nourished by the world’s third-longest and fourth-largest sediment-flux river^9^, extensive tidal flats developed in the Yangtze Estuary over the Holocene^10^. These tidal flats are in decline, in part because the estuary is adjacent to the largest economic center in China that includes 100 million people and contributes 20% to the national Gross Domestic Product (GDP)^4^. High pressure from the dense population and rapid economic development has prompted local governments to enclose and claimed tidal flats as new land resources^11^. The sediment flux of the Yangtze River has dropped by 90% over the past half century due to numerous hydrological projects along the river and its tributaries^12^. Sea-level rise and land subsidence also threaten tidal flats in the estuary, which are predicted to suffer considerable decrease in area or even complete loss in some regions^13^. Because of the increasing tension between the needs of the natural system and socio-economic development in the Yangtze Estuary, many studies have focused on the dynamics of tidal flats or land claim, at different temporal and spatial scales^14^,^15^. By combining the tidal flat and land claim datasets, researchers could assess the impact of land claim on tidal flats^16^ and provide basic data to guide policy making.

The shoreline or waterline, which is the physical interface of land and water, changes its position continually through time because of sediment transport and tidal cycles^8^. Satellites provide near-continuous monitoring of many global shorelines, and the use of Landsat Thematic Mapper (TM)^17^ and Enhanced Thematic Mapper (ETM+) imagery provides a reasonable trade-off between temporal and spatial coverage and between spectral resolution and cost. A range of methods have been developed to establish the location of the waterline, of which density slice, using single or multiple bands and multispectral classification, has been the most common technique^18^. Murray *et al.*, for example, applied the Normalized Difference Water Index (NDWI) to determine the shoreline and manually set thresholds to each image to map the tidal flats of the Yellow Sea^19^. In another study, Xiao *et al.* developed the algorithm ‘LSWI > NDVI’ (LSWI, Land Surface Water Index, also called NDWI; NDVI, Normalized Difference Vegetation Index) to identify persistent and seasonally flooded water bodies^20^. Based on the logical combination of indices, the subjective manual thresholding process could be avoided, and an automatic and reproducible method could be developed to determine shoreline position^18^. However, the random interference factors (e.g., the dynamics of tidal rhythm, water turbidity, and high water content of the substrate) make it difficult to determine tidal flat area. In this study, we mined the temporal series of Landsat images and produced improved maps of water, mudflats, and vegetation in the coastal zone of the Yangtze Estuary. This pilot study demonstrates the potential application of an automatic shoreline algorithm for mapping tidal flats at a large spatial scale.

Based on Landsat imagery (path 118/row 38) covering the Yangtze Estuary, we aimed to: (1) develop a processing tool with simple and robust algorithms for the identification of water, land, and vegetation; (2) document the spatial-temporal dynamics of tidal flats (vegetated saltmarsh and barren mudflat) and land claim from 1984 to 2014 in two areas of the estuary (Shanghai and Jiangsu) that differ in riverine sediment supply and coastal management patterns; and (3) discuss the potential drivers of the spatial-temporal dynamics of tidal flats.

## Results

### Dynamics of tidal flats and land claim in the Yangtze Estuary

**Figure 1:**
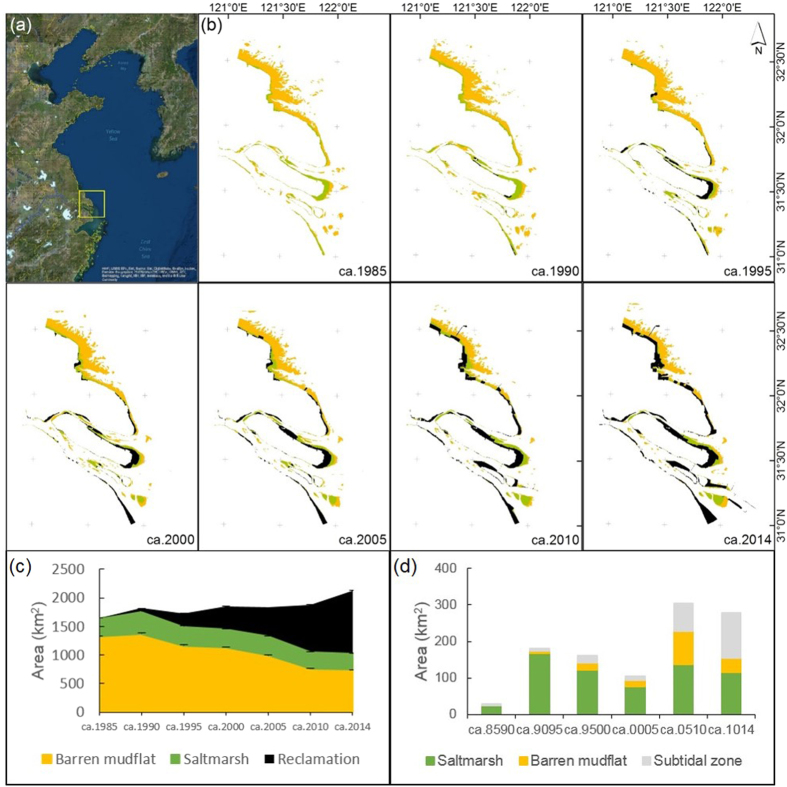
The spatial and temporal dynamics of tidal flats and land claim in the Yangtze Estuary. Processed by ArcGIS 10.1 and ENVI 5.2 (**a**) Location of the Yangtze Estuary (yellow box) in relation to the Yellow Sea. Basemap Source: Esri, DigitalGlobe, GeoEye, i-cubed, Earthstar, Geographics, CNES/Airbus DS, USDA, USGA, AEX, Getmapping, Aerogrid, IGN, IGP, swisstopo, and the GIS User Community. Available at: http://services.arcgisonline.com/ArcGIS/rest/services/World_Imagery/MapServer. (**b**) The encroachment of land claim onto barren mudflat and saltmarsh during the past three decades. Same legend as (**c**). (**c**) Change in the areas of barren mudflat, saltmarsh, and claimed land. The estimated area of the three land classes and 95% confidence intervals. (**d**) Change in the area of enclosed habitats. ca. 8590: ca. 1985–1990, ca. 9095: ca. 1990–1995, ca. 9500: ca. 1995–2000, ca. 0005: ca. 2000–2005, ca. 0510: ca. 2005–2010, ca. 1014: ca. 2010–2014.

In the past three decades, the total area of tidal flats in the estuary has decreased by 36% (from 1647 km^2^ in ca. 1985 to 1047 km^2^ in ca. 2014), comprising a 38% loss of saltmarshes and a 31% loss of barren mudflats (Fig. 1a–c). The total area of land claim has reached 1077 km^2^, a value that exceeds the area of the remaining tidal flats. Together with expanding land claim, the location of sea dikes has moved seaward from saltmarshes to barren mudflats and then to subtidal zones in some regions. For example, saltmarshes represented 87% of the enclosed region in the 1980s but only 42% in the 2010s, and subtidal zones represented 13% of the enclosed region in the 1980s and 45% in the 2010s (Fig. 1d).

### Comparing the dynamics of tidal flats and land claim in Shanghai and Jiangsu

**Figure 2:**
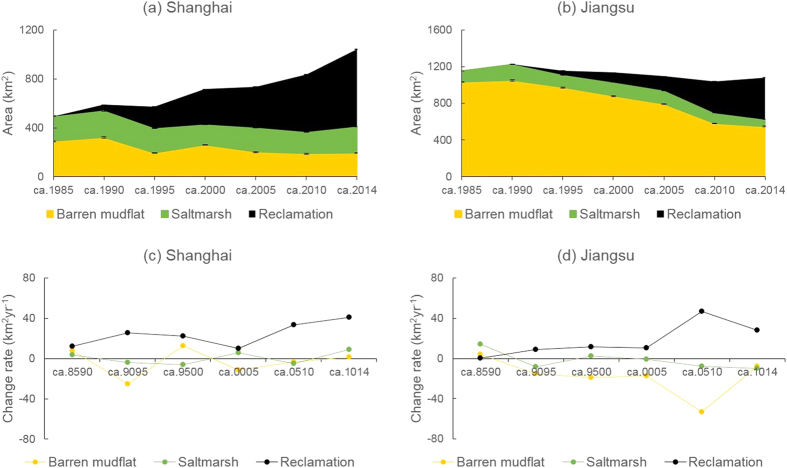
Changes in the areas and rates of tidal flats (saltmarsh and barren mudflat) and land claim in Shanghai and Jiangsu. (**a**) Changes in areas (and 95% confidence intervals) in Shanghai. (**b**) Changes in areas (and 95% confidence intervals) in Jiangsu. (**c**) Rates of area change in Shanghai. (**d**) Rates of area change in Jiangsu. The labels of horizontal axis are the same as Fig. 1d.

Although tidal flats in both areas have experienced large-scale land claim, tidal flat dynamics were different in Shanghai and Jiangsu. In Shanghai, the total area of tidal flats deceased by 16% (from 495 km^2^ in ca. 1985 to 417 km^2^ in ca. 2014) over the past three decades, including a 14% decline in saltmarshes (from 265 km^2^ in ca. 1985 to 227 km^2^ in ca. 2014) and a 17% decline in barren mudflats (from 230 km^2^ in ca. 1985 to 190 km^2^ in ca. 2014) (Fig. 2a). In contrast, tidal flats in Jiangsu suffered a 45% loss (from 1153 km^2^ in ca. 1985 to 630 km^2^ in ca. 2014), including a 59% loss of saltmarshes (from 155 km^2^ in ca. 1985 to 63 km^2^ in ca. 2014) and a 43% loss of barren mudflats (from 998 km^2^ in ca. 1985 to 567 km^2^ in ca. 2014) (Fig. 2b). During the same period, the total area of land claim was 626 km^2^ in Shanghai and 451 km^2^ in Jiangsu. The area of saltmarshes and barren mudflats declined by an annual average of 1.3 and 1.3 km^2^, respectively, in Shanghai and by 3.1 and 14.4 km^2^, respectively, in Jiangsu. The enclosed area increased by an annual average of 20.9 km^2^ in Shanghai and 15.0 km^2^ in Jiangsu. In Shanghai, the rate of land claim increased from ca. 1990 to 1995 and from ca. 2005 to 2014 (Fig. 2c), whereas in Jiangsu, it increased from ca. 2005 to 2010 (Fig. 2d).

## Discussion

### Influence of sediment deposition on the dynamics of tidal flats

This study indicates that over one-third of the tidal flats in the Yangtze Estuary have disappeared over the past three decades, and that saltmarshes have suffered greater losses than barren mudflats. Although the total enclosed area was larger in Shanghai than in Jiangsu, tidal flats have been lost at a much faster rate in Jiangsu than in Shanghai. This might be related to differences in sediment deposition. Deposition of sediments in the estuary is a major driver for the growth or maintenance of tidal flats, and sediment starvation will eventually cause coastal erosion or stagnation of deltaic growth^21^. The Yangtze River experienced a sharp drop in sediment flux (decline of 90%) over the past half century due to construction of dams and waterworks on the river and its tributaries^11^,^12^ (Supplementary Fig. S2). In the Yangtze Estuary, bypass runoff of the north branch declined from 25% to 1% over the past century because the trunk stream of the river has moved continually southward^22^. Moreover, re-suspension of sediments from eroding coasts and subaqueous areas might also contribute to the growth of the tidal flats along the south branch^23^. Thus, erosion of the tidal flats was greater in Jiangsu (along the north branch) than in Shanghai (along the south branch).

To accelerate sediment deposition in the estuary, the Shanghai government has adopted a series of siltation measures, including the planting of dense-rooted Smooth Cordgrass (*Spartina alterniflora*) on the tidal flats, the construction of dikes, and the pumping of sediments from the shallow seabed. Since its introduction in the mid-1980s, Smooth Cordgrass has spread rapidly (it now covers nearly half of the saltmarshes in the Yangtze Estuary) and has changed the structure and functioning of local ecosystems^24^. The Deep Waterway Project (1997–2005) dredged the central portion of the south branch of the Yangtze Estuary from a depth of −6.5 m to −12.5 m and deposited millions of tons of sediment onto nearby tidal flats^25^. Thus, artificial siltation might mitigate the reduction of tidal flats caused by accelerated land claim. However, this will also affect the structure and stability of the soft-bottom ecosystem, and the long-term ecological effects are unknown.

### Escalating tidal flat claim in the Yangtze Estuary

Over the past three decades, the land claim area in the Yangtze Estuary has increased and the area enclosed now exceeds the remaining area of tidal flats. With advancements in techniques and equipment, land claim has expanded seaward, from saltmarshes to barren mudflats, and now has encroached on the subtidal zone in some regions. Meanwhile, increases in both population density and per capita GDP in Shanghai (Supplementary Fig. S3) have driven the demand for more land, which may result in increased pressure to enclose land in Jiangsu. In the face of this pressure for new land, there is a need for local governments to formulate policies for the management of tidal flat resources^5^.

In Shanghai, the rate of land claim increased in two periods, i.e., ca. 1990–1995 and ca. 2005–2014. The former period was mainly influenced by the ‘enclosing the sea for farmlands’ strategy, the development of an Economic Development Zone, and national arable land protection policies. To curb the loss of arable lands to non-agricultural uses, the 1999 Land Administration Law stressed a dynamic balance farmland policy (no net loss in farmland), and the National Land Management Law and the National Land Use Plan (2006–2020) proposed a minimal area of 120 million hectares of national farmland. These strategies encouraged local governments to enclose tidal flats for arable land compensation. The Twelfth Five-Year National Plan in 2010 aimed to develop the marine economy; it envisioned Shanghai as an international shipping center and the claiming of 587 km^2^ of tidal flats till 2020. In Jiangsu, land claim began in ca. 1995 under the development strategy of ‘Marine Sudong’ and increased suddenly from ca. 2005–2010. Functioning as a key national land reserve zone (“Twelfth Five-Year National Plan”), Jiangsu launched the ‘Million Land Project’, which will enclose a further 1,800 km^2^ of tidal flats according to the ‘Jiangsu Marine Function Zoning (2006–2020)’.

Sea-level rise and its impacts on low-lying coastal areas has attracted world-wide attention^13^,^26^, and some developed countries are adopting ‘managed realignment’ sea defense policies^27^ rather than promoting land claim for agricultural and industrial purposes with reinforcing sea walls, which has accelerated wetland loss in China^5^. Even though the construction of sea walls and embankments is considered a form of sea defense, these conventional engineering solutions are not sustainable because of the high maintenance costs, potential for land subsidence, and the blockage of natural sediment deposition^28^. Tidal flats, in contrast, and especially those with vegetated saltmarsh, provide natural sea defense in coastal regions because they can slow water flow, buffer tide and wave energy, and bind sediments by roots, thereby reducing the capacity for erosion. However, tidal flat in the Yangtze Estuary is likely to decrease substantially in the medium-term (2050) and long-term (2100) because of the combined effects of sea-level rise, sediment starvation, and land subsidence^13^. Thus, maintaining the valuable ecosystem services provided by tidal flats will require that rate of land claim does not exceed the rate of accretion of tidal flats, thereby ensuring the regeneration of tidal flats^29^. It is now imperative that we develop sound management policies based on the dynamics of tidal flats so that there is at least ‘no net loss’ in coastal regions^5^.

### The improvement and uncertainty of monitoring horizontal dynamics of tidal flats

A variety of data sources are available for monitoring tidal flat dynamics^8^. These sources differ in many aspects, including spatial and temporal coverage, resolution, production and acquisition costs. Many previous studies in the Yangtze Estuary used coastal maps and charts or conducted ground surveys to study the geomorphologic changes of tidal flats^12^,^15^. Although these methods provide high spatial resolution, they are not appropriate for large-scale and time-series monitoring. Satellites now provide nearly continuous monitoring of many of the world’s shorelines^17^, and Landsat TM/ETM+ images have been increasingly popular because of their high temporal coverage (the revisit period is 16 days), high spatial coverage (nearly global), medium spectral resolution (30 m), and open access^30^. Also, the ‘waterline technique’ (equivalent to the detection of shorelines in coastal zones) based on satellite remote sensing is potentially a highly effective tool for studying tidal flats^31^. Monitoring the horizontal dynamics of tidal flats involves three main steps: developing an algorithm to determine shoreline position; determining tidal flat area at specific periods; and determining the changes in tidal flat area over time^8^. Here, we discuss how our methods have improved these three steps. We also discuss the uncertainty associated with some of our findings.

In the past, shorelines were identified based on visible coastal imagery or on the intersection of a tidal datum with the coastal profile. Based on satellite imagery and image-processing techniques, shorelines can now be identified from digital coastal images that are not necessarily visible to the human eye^8^. As for the reflectance of wavelengths of light, the NIR and SWIR bands have been commonly used to detect the location of shorelines; because these bands are strongly absorbed by water bodies, water bodies show a distinctively low spectral reflectance in this range^32^. However, the NIR band is sensitive to turbid water^33^, and the SWIR band is influenced by remnant surface water on mudflats^32^. Multi-band indices were subsequently developed to take advantage of the reflectance of different wavelengths of light^34^. LSWI (also called NDWI), which includes the NIR and green band, is widely used to delineate open water from terrestrial environments^18^,^35^. When mapping tidal flats of the Yellow Sea, Murray *et al.* applied NDWI and manually set the threshold value for each image to determine the shorelines^36^. Because NDVI can reduce the effect of suspended sediment near shorelines by using a ratio of NIR to visible band^37^, Xiao *et al.* developed an algorithm ‘LSWI > NDVI’ to identify persistent and seasonally flooded water bodies^20^. Because of the high water content of tidal flats, however, the algorithm ‘LSWI > NDVI’ cannot distinguish a mudflat from seawater; we therefore modified this algorithm to ‘LSWI > NDVI + 0.5’, i.e., we increased the threshold for water content. The logical combination of indices also helps researchers develop an automatic algorithm and avoid the use of a subjective and time-consuming manual threshold^18^. With the improved shoreline algorithm ‘LSWI > NDVI + 0.5’, we could identify tidal flats automatically and reproducibly. The use of a fixed threshold, however, may result in some errors in the determination of shorelines because the reflectance of mudflats and seawater has a continuous distribution.

To determine the distribution and extent of tidal flats at a large scale, mapping methods must solve two problems: (a) the scarcity of observed data for tide height; and (b) the variation in the position of the high-tide shoreline^19^. Murray *et al.* used a tide model to generate tide height for each image, and screened for images that were in the highest and lowest 10% of the tidal range when cloud coverage was <30%^19^. This kind of image screening could discard a substantial amount of useful pixel information. Determining shoreline is also limited by many interfering factors, such as wind flows, water currents, particle grain sizes, moisture content, local slope, seawater turbidity, dune juxtapositions, and shadows^18^,^19^, such that 13.2% of the studied coastline cannot be mapped^19^. Given the lack of actual tide data, we used Landsat imagery and assessed all possible pixels within a 5-yr period to determine the shorelines at the lowest tides. This process could also minimize the effects of the interfering factors listed above. Because Landsat sensors cannot detect waterlines under dense saltmarsh vegetation, we substituted the highest tide shorelines with artificial shorelines (which were based on visual examination of Landsat images) as the shoreward boundaries of tidal flats. This process enables us to estimate the largest area of saltmarshes. The accuracies of our maps were acceptable.

Given the lack of actual tide height data, it is difficult to decide whether the estimates of changing area are caused by a different tide range at each time step or by an actual change in tidal flat area. In an attempt to solve this problem, we used tide tables^38^ to assign tide heights to each Landsat image according to its acquisition date and time (Zhongjun Port in Shanghai). Then we compared the differences of tide range between Landsat imagery and tide tables. The results showed that the average maximum tidal range was 448.7 ± 6.2 cm for tide tables and 334.5 ± 23.7 cm for Landsat imagery (Supplementary Table S4), which indicates that the application of all Landsat images within a 5-yr period was insufficient to detect the actual maximum extent of the tidal flats. If we focused on the change in tidal flat area around the tidal range of 334.5 cm, however, the standard error was 23.7 cm, which means that tides contributed 7% to the error in our estimates of tidal flat area.

## Methods

### Study region

Located at the junction of the Yellow Sea and East China Sea (Fig. 1a), the Yangtze Estuary is one of the largest estuarine alluvial wetlands in the world^13^. During the Holocene, about 11.6 × 10^11^ t of sediment accumulated in the estuary and proximal subaqueous delta, and another 5.4 × 10^11^ t was transported southward off the Zhejiang and Fujian coasts into the Taiwan Strait^39^. Tides in this estuary are typically semidiurnal, with a mean tidal range of 2.0–3.1 m and a maximum tidal range of 4.6–6.0 m^40^. The average width of the tidal flats is 1–3 km, and the greatest width exceeds 10 km.

The Yangtze Estuary is divided by Chongming Island into northern and southern branches. The southern branch is the main water channel, carrying 90% of the total amount of runoff and sediment into the sea (Supplementary Fig. S1a)^11^. Two administrative regions govern the Yangtze Estuary: Shanghai City on the southern bank and Jiangsu Province on the northern bank. According to the presence or absence of vegetation, tidal flats can be divided into saltmarshes and barren mudflats (Supplementary Fig. S1b–f). The main native plant species in the saltmarshes of the Yangtze Estuary are reed (*Phragmites australis*) and bulrush (*Scirpus spp.*). Over the past two decades, introduced Smooth Cordgrass has colonized about half of the vegetated tidal flats in the Yangtze Estuary^24^.

### Data acquisition and pre-processing

By downloading data from the USGS Data Center (http://glovis.usgs.gov/), we collected all available Landsat TM and ETM+ images (651 images) for the path 118/row 38 from 1984 to 2014, including all level 1 terrain-corrected products (L1T), Level 1 Systematically Corrected (L1G), and Systematically Terrain Corrected (L1Gt)^41^. Through visual screening, we excluded three images with remarkable deviations.

**Figure 3:**
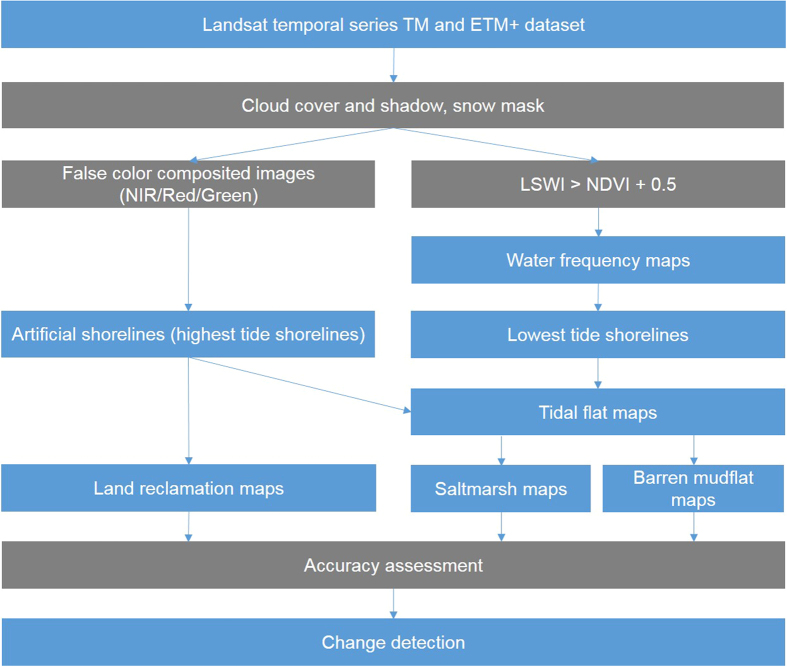
Flow chart used for the development of tidal flat and land claim maps for the Yangtze Estuary.

We conducted atmospheric correction on the Landsat images to acquire the surface reflectance data using LEDAPS software^42^, which has been widely used in Landsat data preprocessing^43^ (Fig. 3). Then we generated a time series of Landsat NDVI, LSWI, and Normalized Difference Snow Index (NDSI) datasets.


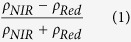



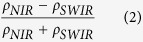



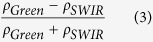


where *ρ*_*Green*_, *ρ*_*Red*_, *ρ*_*NIR*_ and *ρ*_*SWIR*_ are the surface reflectance values of Band 2 (Green, 0.53–0.61 μm), Band 3 (Red, 0.63–0.69 μm), Band 4 (NIR, 0.76–0.90 μm), and Band 5 (SWIR, 1.55–1.75 μm) in the Landsat TM/ETM+ sensors.

To minimize interference from clouds and other weather factors, we generated three filters^44^: (1) ETM+ scan-line corrector (SLC)-off pixels, excluded according to their metadata; (2) Clouds and cloud shadow were detected with the Fmask package^45^; (3) Snow cover was identified with the thresholds NDSI > 0.40 and NIR > 0.11^46^. All bad observations were then removed from each image.

### Maps of land claim

We selected one cloudless Landsat image for each period: ca. 1985 (1984–1987); ca. 1990 (1988–1992); ca. 1995 (1993–1997); ca. 2000 (1998–2002); ca. 2005 (2003–2007); ca. 2010 (2008–2011); and ca. 2014 (2012–2014) (Supplementary Table S1). Given the complex structures, we delineated artificial shorelines through visual interpretation based on the false color-composited images (musR/G/B = NIR/Red/Green) (Supplementary Fig. S1b). Then we produced land claim maps by stacking images of artificial shorelines of successive periods.

### Maps of tidal flats (water, mudflat, and vegetation)

**Figure 4:**
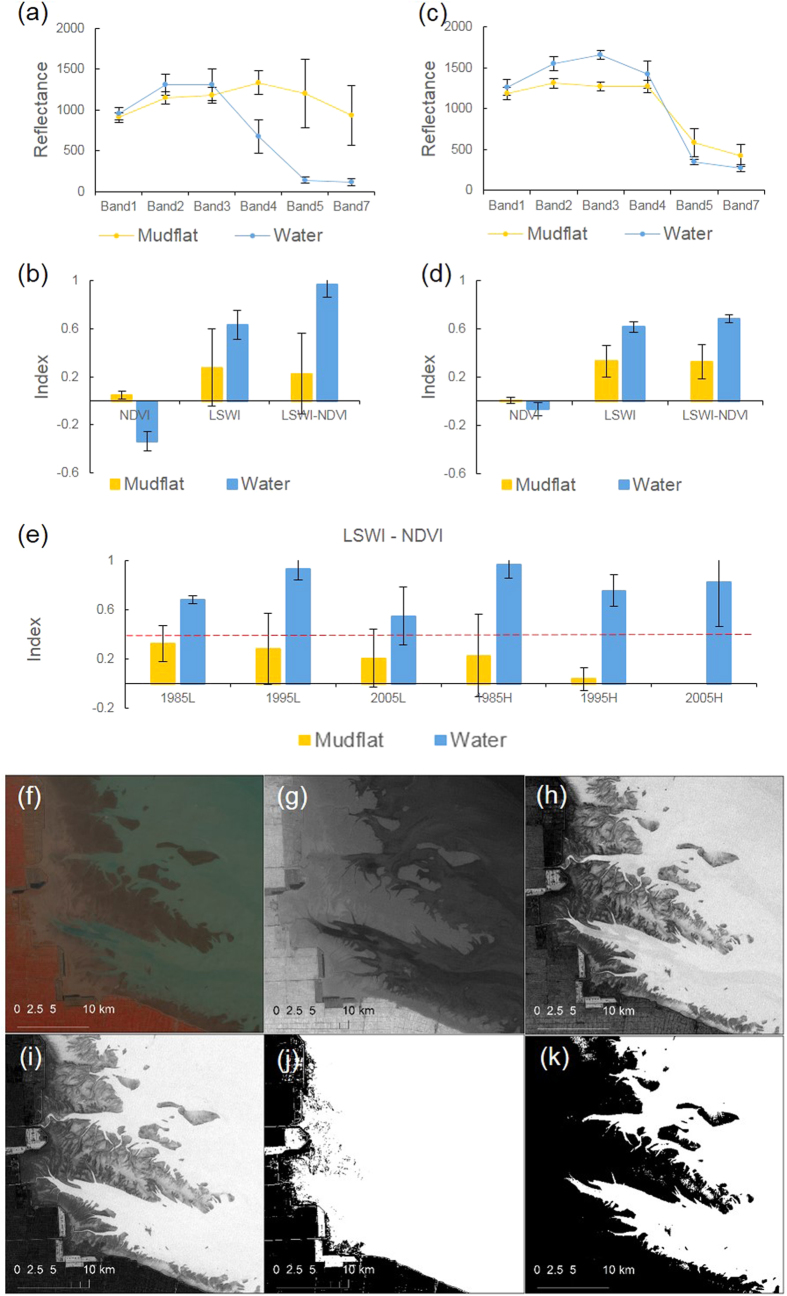
Information used to develop an algorithm to determine shorelines in the Yangtze Estuary. Processed by ArcGIS 10.1 and ENVI 5.2 (**a**) The reflectance difference of barren mudflat and seawater during low tide. Landsat image LT51180381985052HAJ00 is used as an example. (**b**) The differences of NDVI, LSWI, and “LSWI - NDVI” between barren mudflat and seawater during low tide. (**c**) The reflectance difference of barren mudflat and seawater during high tide. Landsat image LT51180381985324HAJ00 is used as an example. (**d**) The NDVI, LSWI, and “LSWI − NDVI” differences of barren mudflat and seawater during high tide. (**e**) The use of a fixed threshold of 0.5 for “LSWI − NDVI” images for distinguishing barren mudflat from seawater. 1985L: the lowest tide shoreline in 1985, 1995L: the lowest tide shoreline in 1995, 2005L: the lowest tide shoreline in 2005, 1985H: the highest tide shoreline in 1985, 1995H: the highest tide shoreline in 1995, 2005H: the highest tide shoreline in 2005. (**f**) False color composite Landsat image (musR/G/B = NIR/Red/Green) of LT51180381985052HAJ00. Source: the U.S. Geological Survey. Available at: http://www.usgs.gov. (**g**) NDVI. (**h**) LSWI. (**i**) LSWI − NDVI. (**j**) LSWI > NDVI. White indicates water, Black indicates non-water. (**k**) LSWI > NDVI + 0.5.

To develop a shoreline algorithm, we screened for images with either the highest or lowest shoreline in 1985, 1995, and 2005 and conducted a complete signature analysis of barren mudflat and seawater (Supplementary Table S2). The results showed that mudflats reflect higher amounts of energy in NIR and SWIR than seawater at high tide but that reflectance did not strongly differ between barren mudflat and seawater at low tide (Fig. 4a,c). Because LSWI and NDVI showed the opposite trend between mudflat and seawater, subtracting LSWI from NDVI can highlight their difference (Fig. 4b,d,g–i). Xiao *et al.* developed an algorithm ‘LSWI > NDVI’ to identify persistent and seasonally flooded water bodies^20^, but this algorithm cannot distinguish between mudflat and seawater because of the high water content of mudflats (Fig. 4f,j). Based on the signature analysis of ‘LSWI − NDVI’ images, we found that a threshold of 0.5 could separate most mudflat areas from seawater areas (Fig. 4e). Thus, we developed the algorithm ‘LSWI > NDVI + 0.5’ and applied a band math tool to determine the area occupied by seawater (Fig. 4k).

To deal with the low probability of capturing an image of low tide shorelines during satellite passage time, we stacked all images within a 5-yr period to develop water frequency maps. Considering the water frequency of seawater is 1, the water frequency of tidal flats should be scaled between 0–1 due to the tide cycle. Since some random factors may reduce the water frequency of seawater and misclassify seawater into tidal flats, we adjusted the water frequency range of tidal flats to 0–0.75. Then, we used data delineation tools to extract the observed lowest tide shorelines. Because the high tide shoreline under dense vegetation cannot be detected through Landsat sensors, the area of saltmarshes could be underestimated. In these cases, we visually delineated artificial shorelines and considered them as the shoreward boundaries of tidal flats. We then determined the area between the artificial and lowest tide shorelines in each period, and this area was considered the maximum exposed extent of the tidal flats. Finally, we assessed the accuracies of our maps, and the outcomes were acceptable. Then we generated tidal flat maps based on the area between the lowest and highest tide shorelines. Barren land was defined as ‘NDVI < 0.1’ and vegetated land as ‘NDVI > 0.1’^47^. Based on this threshold, we further divided tidal flat maps into saltmarsh and barren mudflat maps.

### Accuracy assessment

We independently validated our maps in each period by following the validation steps referring to Olofsson *et al.*^48^,^49^. We used time series Landsat imagery and a visual approach for validation. We also used photographs that were geographically positioned in the field using global positioning system or downloaded from the Global Geo-Referenced Field Photo Library (http://www.eomf.ou.edu/photos), and high spectral resolution images in Google Earth^TM^ for the validation.

To validate our estimates of the areas of the three land classes (barren mudflat, saltmarsh, and claimed land), we screened one Landsat image for each period at low tide at a time during the growing season (May to October) when there were no clouds (Supplementary Fig. S1c–f). We used stratified random sampling and proportional sample allocation to select the subset of spatial points. By requiring a standard error of 0.01 for the estimated overall accuracy and a standard deviation of 0.7 for each class, we calculated a total sample size of 2000 for maps of each period (Olofsson 2014, Eq. (13))^49^ (Supplementary Fig. S1a and Table S2). Accordingly, we generated random points in each class and made sample units as round buffers of the points (diameter 60 m).

Based on false color composite images (musR/G/B = NIR/Red/Green), we used a visual approach to create the reference classification. Because tidal flat maps were produced by stacking all images within a 5-yr period, and only one image for reference classification in each period, the samples classified as saltmarsh or mudflat in maps could be labeled as pure or mixed seawater in the reference classification, simply due to the tide cycle. Thus, in the reference classification, we deleted the pure seawater samples, and assigned the mixed seawater samples to the corresponding classes.

Based on the sample counts of mapping and reference classification, we built an error matrix and calculated overall, user’s, and product’s accuracies (Olofsson 2014, Eqs (1, 2, 3))^49^ (Supplementary Table S3). By replacing the sample counts in the error matrix with the estimated area proportions (Olofsson 2014, Eq. (4))^49^, we built an estimated error matrix and calculated the estimators of accuracies and their 95% confidence intervals (Olofsson 2014, Eqs (5–7))^49^. Finally, we calculated the estimated proportion of area and estimated area for each class (Olofsson 2014, Eqs (8, 10, 11))^49^. The estimated overall accuracy across the sample set was >91%. The estimated user’s accuracy was >96%, >64%, and >96% for barren mudflat, saltmarsh, and claimed land, respectively. The estimated producer’s accuracy was >81%, >92%, and >94% for barren mudflat, saltmarsh, and land claim, respectively (Supplementary Table S3).

All of the above processing was undertaken with IDL 8.4, ArcGIS 10.1 and ENVI 5.2 software.

### Analyses of spatial–temporal dynamics of tidal flats and land claim

For land claim maps showing recent increases in the area of enclosed land, we directly obtained the increase in area between periods. Tidal flat maps showed the remaining area, and then the change in the area of tidal flats was calculated by subtracting the remaining area of successive periods. The rates of change in the areas of tidal flats and land claim were determined by dividing the total change in area by the number of years in each interval. We intersected the saltmarsh or barren mudflat maps with land claim maps and then calculated the enclosed saltmarsh and barren mudflat area in each period. If the claimed lands were neither on saltmarsh nor on barren mudflat, we defined the enclosed habitat as subtidal zone.

## Additional Information

**How to cite this article**: Chen, Y. *et al.* Land claim and loss of tidal flats in the Yangtze Estuary. *Sci. Rep.* **6**, 24018; doi: 10.1038/srep24018 (2016).

## Supplementary Material

Supplementary Information
