# Supplementary material for: Land claim and loss of tidal flats in the Yangtze Estuary
Source: Sci Rep. 2016 Apr 1;6:24018. doi: 10.1038/srep24018 (PMC4817514; doi:10.1038/srep24018)
Supplement: Supplementary Information [file srep24018-s1.doc]

**Land claim and loss of tidal flats in the Yangtze Estuary**

Ying Chen^1,2^, Jinwei Dong^2^, Xiangming Xiao^1,2^, Min Zhang^3^, Bo Tian^3^,

Yunxuan Zhou^3^, Bo Li^1^, Zhijun Ma^1*^

1 Coastal Ecosystems Research Station of Yangtze Estuary, Ministry of Education Key Laboratory for Biodiversity Science and Ecological Engineering, Institute of Biodiversity Science, Fudan University, Shanghai, 200438, China

2 Department of Microbiology and Plant Biology, Center for Spatial Analysis, University of Oklahoma, Norman, OK 73019, USA

3 State Key Laboratory of Estuary and Coastal Research, East China Normal University, Shanghai, 200062, China

^*^ Correspondence to zhijunm@fudan.edu.cn


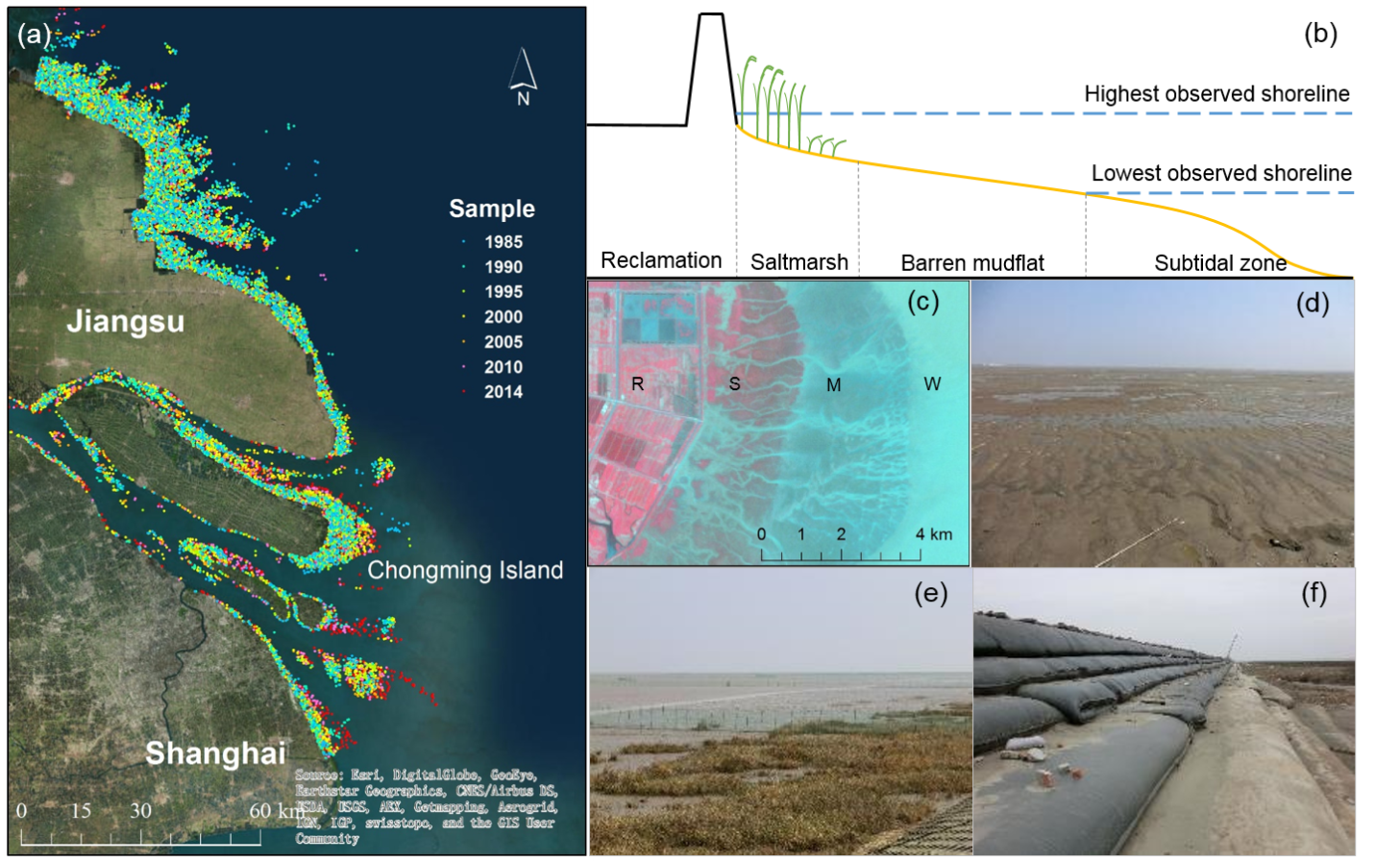


Supplementary Fig. S1. Study region and habitat types of tidal flats. Processed by ArcMap 10.3.1. (a) The Yangtze Estuary and the locations of samples used for the accuracy assessment in different periods are indicated by different colors. Source: World Imagery, Esri, DigitalGlobe, GeoEye, i-cubed, Earthstar, Geographics, CNES/Airbus DS, USDA, USGA, AEX, Getmapping, Aerogrid, IGN, IGP, swisstopo, and the GIS User Community. Available at: http://services.arcgisonline.com/ArcGIS/rest/services/World_Imagery/MapServer. (b) Schematic diagram of tidal flats. (c) Spectral characteristics of tidal flat habitats on a Landsat image. R: Reclaimed land, S: Saltmarsh, M: Barren mudflat, W: Seawater. Source: False color composite Landsat image (musR/G/B=NIR/Red/Green) of LT51180381995224HAJ00 of the U.S. Geological Survey. Available at: http://www.usgs.gov. (d) Barren mudflat. (e) Saltmarsh. (f) Newly built sea dike. All photographs were taken by Y.C.

Supplementary Table S1. Summary of Landsat images used for determining the artificial shorelines and assessing the accuracies of our maps.

| Period | Satellite image | | Total area of three land classes (km^2^)/Sample count | | |
| --- | --- | --- | --- | --- | --- |
|  | Sensor | Acquisition date | Mudflat | Saltmarsh | Reclaimed land |
| 1985 | TM | 1986-05-15 | 1227.8/1491 | 419.6/509 | 0/0 |
| 1990 | TM | 1989-08-11 | 1248.1/1346 | 522.0/554 | 47.5/100 |
| 1995 | TM | 1995-08-12 | 1138.0/1317 | 362.2/419 | 227.9/264 |
| 2000 | TM | 2002-07-30 | 1073.1/1160 | 388.6/420 | 388.8/420 |
| 2005 | ETM+ | 2005-06-12 | 949.6/1037 | 387.0/423 | 494.1/540 |
| 2010 | ETM+ | 2008-07-06 | 624.1/665 | 454.2/484 | 798.4/851 |
| 2014 | ETM+ | 2013-05-01 | 756.9/713 | 289.8/273 | 1077.4/1014 |

Supplementary Table S2. Summary of Landsat images used for developing the algorithm to determine the location of shorelines. Low: Low tide, High: High tide.

| Satellite image | | | Tide condition | Sample area (km^2^)/ Sample count | |
| --- | --- | --- | --- | --- | --- |
| Sensor | Acquisition date | Cloud cover (%) |  | Seawater | Mudflat |
| TM | 1985-02-11 | 9 | Low | 334.9/20 | 61.7/18 |
| TM | 1985-11-12 | 0 | High | 306.4/16 | 178.6/18 |
| TM | 1995-05-08 | 0 | Low | 413.4/16 | 46.5/14 |
| TM | 1995-07-11 | 24 | High | 507.8/22 | 140.4/23 |
| ETM+ | 2005-06-12 | 0 | Low | 235.0/19 | 1003.6/0 |
| ETM+ | 2005-09-16 | 0 | High | 261.0/23 | 82.5/36 |

Supplementary Table S3. Error matrices and users’, producer’s, and overall accuracies of maps in seven periods. Class 1: Barren mudflat, Class 2: Saltmarsh, Class 3: Reclaimed land. Each table includes two parts: the first part used sample counts to calculate the accuracies, and the second part used estimated area proportions to calculate the estimated accuracies and their 95% confidence intervals. Rows are mapping categories, and columns are reference categories.

| Class | 1 | 2 | 3 | Total | Area proportion | User’s accuracy | Producer’s accuracy | Overall accuracy |
| --- | --- | --- | --- | --- | --- | --- | --- | --- |
| 1985 |  |  |  |  |  |  |  |  |
| 1 | 1233 |  |  | 1233 | 0.75 | 1.00 | 0.92 | 0.94 |
| 2 | 104 | 364 | 0 | 468 | 0.25 | 0.77 | 1.00 |  |
| 3 |  |  |  | 0 |  |  |  |  |
| Total | 1337 | 364 |  | 1701 |  |  |  |  |
| Estimation |  |  |  |  |  |  |  |  |
| 1 | 0.75 | 0.00 | 0.00 | 0.75 | 0.80 | 1.00±0.00 | 0.93±0.01 | 0.94±0.01 |
| 2 | 0.06 | 0.20 | 0.00 | 0.25 | 0.20 | 0.78±0.04 | 1.00±0.00 |  |
| 3 |  |  |  |  |  |  |  |  |
| Total | 0.80 | 0.20 | 0.00 | 1.00 |  |  |  |  |

| Class | 1 | 2 | 3 | Total | Area proportion | User’s accuracy | Producer’s accuracy | Overall accuracy |
| --- | --- | --- | --- | --- | --- | --- | --- | --- |
| 1990 |  |  |  |  |  |  |  |  |
| 1 | 557 | 2 |  | 559 | 0.69 | 1.00 | 0.82 | 0.89 |
| 2 | 120 | 376 | 3 | 499 | 0.29 | 0.75 | 0.99 |  |
| 3 |  |  | 100 | 100 | 0.03 | 1.00 | 0.97 |  |
| Total | 677 | 378 | 103 | 1158 |  |  |  |  |
| Estimation |  |  |  |  |  |  |  |  |
| 1 | 0.68 | 0.00 | 0.00 | 0.69 | 0.75 | 1.00±0.01 | 0.91±0.01 | 0.93±0.01 |
| 2 | 0.07 | 0.22 | 0.00 | 0.29 | 0.22 | 0.75±0.04 | 0.99±0.02 |  |
| 3 | 0.00 | 0.00 | 0.03 | 0.03 | 0.03 | 1.00±0.00 | 0.94±0.07 |  |
| Total | 0.75 | 0.22 | 0.03 | 1.00 |  |  |  |  |

| Class | 1 | 2 | 3 | Total | Area proportion | User’s accuracy | Producer’s accuracy | Overall accuracy |
| --- | --- | --- | --- | --- | --- | --- | --- | --- |
| 1995 |  |  |  |  |  |  |  |  |
| 1 | 1022 | 7 |  | 1029 | 0.66 | 0.99 | 0.96 | 0.96 |
| 2 | 38 | 368 | 6 | 412 | 0.21 | 0.89 | 0.96 |  |
| 3 | 3 | 7 | 234 | 244 | 0.13 | 0.96 | 0.98 |  |
| Total | 1063 | 382 | 240 | 1685 |  |  |  |  |
| Estimation |  |  |  |  |  |  |  |  |
| 1 | 0.65 | 0.00 | 0.00 | 0.65 | 0.68 | 0.99±0.01 | 0.97±0.01 | 0.97±0.01 |
| 2 | 0.02 | 0.19 | 0.00 | 0.02 | 0.20 | 0.89±0.03 | 0.96±0.02 |  |
| 3 | 0.00 | 0.00 | 0.13 | 0.13 | 0.13 | 0.96±0.02 | 0.98±0.02 |  |
| Total | 0.67 | 0.20 | 0.13 | 1.00 |  |  |  |  |

| Class | 1 | 2 | 3 | Total | Area proportion | User’s accuracy | Producer’s accuracy | Overall accuracy |
| --- | --- | --- | --- | --- | --- | --- | --- | --- |
| 2000 |  |  |  |  |  |  |  |  |
| 1 | 1042 | 15 |  | 1057 | 0.58 | 0.99 | 0.93 | 0.95 |
| 2 | 82 | 321 | 5 | 408 | 0.21 | 0.79 | 0.96 |  |
| 3 |  |  | 403 | 403 | 0.21 | 1.00 | 0.99 |  |
| Total | 1124 | 336 | 408 | 1868 |  |  |  |  |
| Estimation |  |  |  |  |  |  |  |  |
| 1 | 0.57 | 0.01 | 0.00 | 0.58 | 0.62 | 0.99±0.01 | 0.93±0.01 | 0.95±0.01 |
| 2 | 0.04 | 0.17 | 0.00 | 0.21 | 0.17 | 0.79±0.04 | 0.95±0.02 |  |
| 3 | 0.00 | 0.00 | 0.21 | 0.21 | 0.21 | 1.00±0.00 | 0.99±0.01 |  |
| Total | 0.61 | 0.17 | 0.21 | 1.00 |  |  |  |  |

| Class | 1 | 2 | 3 | Total | Area proportion | User’s accuracy | Producer’s accuracy | Overall accuracy |
| --- | --- | --- | --- | --- | --- | --- | --- | --- |
| 2005 |  |  |  |  |  |  |  |  |
| 1 | 915 | 17 | 1 | 933 | 0.52 | 0.98 | 0.94 | 0.95 |
| 2 | 63 | 337 | 2 | 402 | 0.21 | 0.84 | 0.94 |  |
| 3 |  | 3 | 521 | 524 | 0.27 | 0.99 | 0.99 |  |
| Total | 978 | 357 | 524 | 1859 |  |  |  |  |
| Estimation |  |  |  |  |  |  |  |  |
| 1 | 0.51 | 0.01 | 0.00 | 0.52 | 0.54 | 0.98±0.01 | 0.94±0.01 | 0.95±0.01 |
| 2 | 0.03 | 0.18 | 0.00 | 0.21 | 0.19 | 0.84±0.04 | 0.94±0.02 |  |
| 3 | 0.00 | 0.00 | 0.27 | 0.27 | 0.27 | 0.99±0.01 | 0.99±0.01 |  |
| Total | 0.54 | 0.19 | 0.27 | 1.00 |  |  |  |  |

| Class | 1 | 2 | 3 | Total | Area proportion | User’s accuracy | Producer’s accuracy | Overall accuracy |
| --- | --- | --- | --- | --- | --- | --- | --- | --- |
| 2010 |  |  |  |  |  |  |  |  |
| 1 | 638 | 2 |  | 640 | 0.33 | 1.00 | 0.82 | 0.91 |
| 2 | 144 | 291 | 19 | 454 | 0.24 | 0.64 | 0.99 |  |
| 3 |  |  | 834 | 834 | 0.43 | 1.00 | 0.98 |  |
| Total | 782 | 293 | 853 | 1928 |  |  |  |  |
| Estimation |  |  |  |  |  |  |  |  |
| 1 | 0.33 | 0.00 | 0.00 | 0.33 | 0.41 | 1.00±0.01 | 0.81±0.02 | 0.91±0.01 |
| 2 | 0.08 | 0.16 | 0.01 | 0.24 | 0.16 | 0.64±0.04 | 0.99±0.01 |  |
| 3 | 0.00 | 0.00 | 0.43 | 0.43 | 0.44 | 1.00±0.00 | 0.98±0.01 |  |
| Total | 0.41 | 0.16 | 0.44 | 1.00 |  |  |  |  |

| Class | 1 | 2 | 3 | Total | Area proportion | User’s accuracy | Producer’s accuracy | Overall accuracy |
| --- | --- | --- | --- | --- | --- | --- | --- | --- |
| 2014 |  |  |  |  |  |  |  |  |
| 1 | 618 | 20 | 6 | 644 | 0.36 | 0.96 | 0.98 | 0.97 |
| 2 | 12 | 244 | 11 | 267 | 0.14 | 0.91 | 0.92 |  |
| 3 | 1 | 1 | 994 | 996 | 0.51 | 1.00 | 0.98 |  |
| Total | 631 | 265 | 1011 | 1907 |  |  |  |  |
| Estimation |  |  |  |  |  |  |  |  |
| 1 | 0.34 | 0.01 | 0.00 | 0.36 | 0.35 | 0.96±0.02 | 0.98±0.01 | 0.97±0.01 |
| 2 | 0.01 | 0.12 | 0.01 | 0.14 | 0.14 | 0.91±0.03 | 0.92±0.03 |  |
| 3 | 0.00 | 0.00 | 0.51 | 0.51 | 0.52 | 1.00±0.01 | 0.98±0.01 |  |
| Total | 0.35 | 0.14 | 0.52 | 1.00 |  |  |  |  |

Supplementary Table S4. Summary of Landsat images used for tide range assessment.

| Time slice | Satellite image number | Detected tide range  (minimum – maximum, mm) | Predicted tide range  (minimum – maximum, mm) | Area of tidal flats (km^2^) |
| --- | --- | --- | --- | --- |
| 1985 | 48 | 59 - 336 | 19 - 482 | 1647.4 |
| 1990 | 67 | 59 - 387 | 23 - 481 | 1770.1 |
| 1995 | 69 | 58 - 364 | 16 - 480 | 1500.2 |
| 2000 | 150 | 66 - 397 | 14 - 480 | 1461.7 |
| 2005 | 160 | 44 - 415 | 16 - 478 | 1336.6 |
| 2010 | 77 | 81 - 399 | 32 - 482 | 1078.3 |
| 2014 | 77 | 64 - 417 | 20 - 477 | 1046.7 |


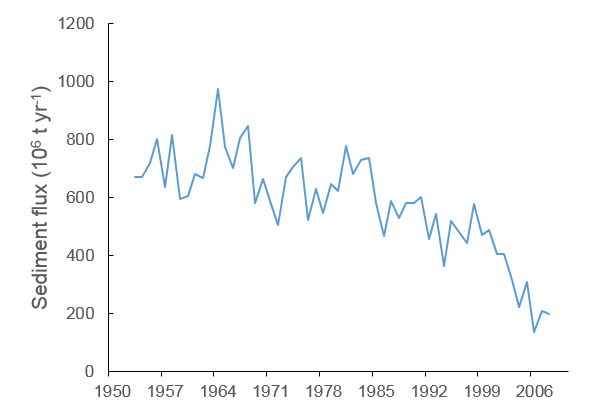


Supplementary Fig. S2. Change of sediment flux in the Yangtze River. The data were measured at Datong, the farthest downstream gauging station ^1^.


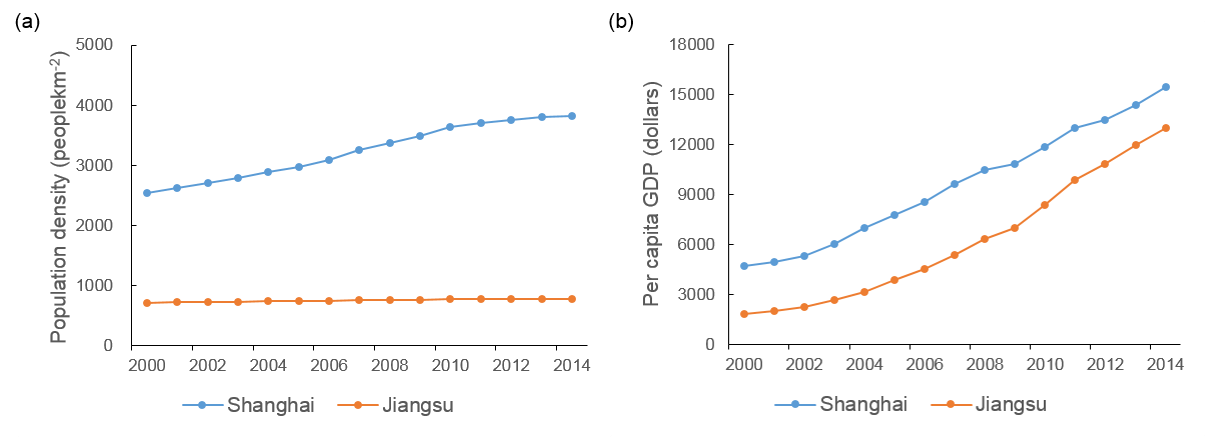


Supplementary Fig. S3. Changes in the population density and in the per capita GDP in Shanghai and Jiangsu. The data were obtained from the National Bureau of Statistics of China^2^.

# **References**

1 Yang, S., Milliman, J. D., Li, P. & Xu, K. 50,000 dams later: erosion of the Yangtze River and its delta. *Global Planet Change* **75**, 14-20 (2011).

2 National Bureau of Statistics of China. Available at: http://data.stats.gov.cn. (2013). Date of access: 17/5/2015.
